# Enhanced down-regulation of ALCAM/CD166 in African-American Breast Cancer

**DOI:** 10.1186/1471-2407-14-715

**Published:** 2014-09-25

**Authors:** Fang Tan, Marina Mosunjac, Amy L Adams, Beverly Adade, Oleyad Taye, Yijuan Hu, Monica Rizzo, Solomon F Ofori-Acquah

**Affiliations:** Aflac Cancer Center and Blood Disorders Service, Department of Pediatrics, Emory University School of Medicine, 2015 Uppergate Drive, Atlanta, GA 30322 USA; Grady Memorial Hospital, Atlanta, GA USA; Department of Pathology and Laboratory Medicine, Emory University School of Medicine, Atlanta, GA USA; Department of Biostatistics and Bioinformatics, Rollins School of Public Health, Emory University, Atlanta, GA USA; Department of Surgery, Division of Surgical Oncology, Emory University School of Medicine, Atlanta, GA USA; University of Pittsburgh, 200 Lothrop Street, Pittsburgh, PA 15261 USA

**Keywords:** ALCAM, African-American, Caucasian, Breast cancer

## Abstract

**Background:**

Variation in tumor biology in African-American (AA) and Caucasian (CAU) women with breast cancer is poorly defined. Activated leukocyte cell adhesion molecule (ALCAM) is a bad prognostic factor of breast cancer yet it has never being studied in the AA population. We tested the hypothesis that ALCAM expression would be markedly lower in cases of AA breast cancer when compared to CAU.

**Methods:**

Cases of breast cancer among AA (n = 78) and CAU (n = 95) women were studied. Immunohistochemical staining was used to semi-quantitatively score ALCAM expression in tumor and adjacent non-tumor breast tissues. Clinico-pathological characteristics including histological type, histological grade, tumor size, lymph node metastasis, estrogen receptor (ER), progesterone receptor (PR), and HER2-neu status were abstracted, and their association with ALCAM expression tested.

**Results:**

Univariate analysis revealed that the level of ALCAM expression at intercellular junctions of primary tumors correlates with histological grade (AA; p = 0.04, CUA; p = 0.02), ER status (AA; p = 0.0004, CAU; p = 0.0015), PR status (AA; p = 0.002, CUA p = 0.034) and triple-negative tumor status (AA; p = 0.0002, CAU; p = 0.0006,) in both ethnic groups. Multivariate analysis demonstrated that ethnicity contribute significantly to ALCAM expression after accounting for basal-like subtype, age, histological grade, tumor size, and lymph node status. Compared to CAU tumors, the AA are 4 times more likely to have low ALCAM expression (p = 0.003).

**Conclusions:**

Markedly low expression of ALCAM at sites of cell-cell contact in primary breast cancer tumors regardless of differentiation, size and lymph node involvement may contribute to the more aggressive phenotype of breast cancer among AA women.

## Background

Breast cancer affects African-American (AA) women at a lower frequency than Caucasian (CAU) women, yet progression of the tumor and mortality from the disease is higher among AA [1]. This difference persists even after taking into account access to care, tumor characteristics, and treatments [2, 3]. There are a few clear explanations for these ethnic disparities [4]. The overwhelming majority of studies aimed at understanding this disparity have focused on socioeconomic and cultural differences, which clearly have significant health consequences across a broad spectrum of diseases, including cancer [2, 5, 6]. On the contrary, there is a paucity of studies on the potential role of heterogeneity in tumor biology in the health disparity of breast cancer in the US.

The discovery of molecular markers that influence prognostic or treatment outcome may help to understand the ethnic disparity in breast cancer in the US [7, 8]. Adhesion molecules tethered at sites of cell-cell contact intimately influence cancer progression and the response to therapy, and are therefore, candidate molecules for understanding this disparity [9–12]. Activated leukocyte cell adhesion molecule (ALCAM/CD166), is an immunoglobulin cell adhesion molecule expressed by neuronal, endothelial, hematopoietic and epithelial cells [13–16]. We showed previously that ALCAM is recruited to sites of cell-cell contact in epithelium [17]. In a study of primary breast cancer tissues and non-neoplastic mammary tissue from the same mastectomies, we discovered that ALCAM mRNA was lower in tumors from patients who had metastases to regional lymph nodes and early mortality [18]. Other studies confirmed that loss of ALCAM function, due to reduced expression and/or protein mislocation is a bad prognostic marker in breast cancer [17–22]. ALCAM coalesces breast cancer cells together in homotypic interactions thus preventing interactions with neighboring endothelium, which may facilitate metastasis [23]. In support of this idea low ALCAM mRNA correlates with the development of skeletal metastasis [24].

Despite the significance of ALCAM in breast cancer this molecule has not previously been studied among AA women. In the current study, we tested the hypothesis that ALCAM expression is low in breast cancer tumors of AA women, and that this phenomenon may contribute to the more aggressive tumor phenotype in this patient population. We found that ALCAM was reduced or completely absent at intercellular junctions of most breast cancer tumors of AA women. On the contrary, the majority of tumors of CAU women had moderate to high ALCAM expression. This ethnic disparity was evident in tumors of similar histological grade, tumor size and lymph node. Thus, loss of ALCAM may contribute to the more aggressive phenotype of breast cancer among AA women.

## Methods

### Patients and tissue blocks

The study protocol was reviewed and approved by Emory University’s Institutional Review Board (IRB) Committee. The consent forms were not required for this study. Patients included in this study were self-reported as AA and CAU diagnosed with invasive breast cancer. A total of 173 cases of invasive breast cancer (78 AA and 95 CAU) in Emory University hospital or Grady Memorial Hospital from 2007 to 2009 were studied. Tumor-related factors (Histological type, histological grade, tumor size and nodal status) were obtained from the independent abstraction of pathology reports. Stage at diagnosis was defined using American Joint Committee on Cancer Stage criteria that are in use during the case ascertainment period (2007–2009) [25]. Stage represents pathologic stage at the time of the first diagnostic procedure confirming invasive breast cancer and was divided into groups (I, II and III/IV). Archived formalin-fixed paraffin-embedded (FFPE) tissue blocks were retrieved and reviewed by the pathologist, who was blinded to ethnicity and other personal characteristics. The ER/PR status and HER2/neu status reported in patient pathology reports were determined by immunohistochemistry (IHC).

### Immunohistochemical analysis

Formalin-fixed paraffin-embedded tissue sections were mounted on superfrost slides and stained using appropriate positive and negative controls as we have described previously [18, 19].

The sections (5 μm) were de-paraffinized, rehydrated and processed for antigen retrieval using Dako Antigen Retrieval Solution. Tissue peroxidases were inactivated by treatment with 3% H_2_O_2_ for 5 min, and the sections pre-treated with antibody diluent solution containing 1% BSA, followed by 40 min incubation at room temperature with primary antibodies for ALCAM (1:40 dilution, Novocastra Laboratories). Labeling was accomplished with biotinylated secondary antibodies and streptavidin-HRP using Biotinylated Link Antibody kit (Dako North America Inc, Carpinteria, CA), AEC substrate chromogen, and counterstained with hematoxylin for 5 min. Sections were mounted with aqueous media, examined using Olympus AX70 microscope and images were recorded with camera (Olympus U-CMAD3 DP70) and software (Olympus DP70/DP30 BW, ver.02.0201.147). Negative control tests were conducted with samples in the absence of primary antibody. Similarly, control paraffin slides with known negative or positive expression ALCAM were tested alongside of unknown samples.

### Evaluation of immunohistochemistry

Stained tissue sections were independently examined in a blinded fashion by two clinical pathologists, who were blinded to clinical information and pathological parameters. ALCAM expression at intercellular junctions (i.e. membranous) and in the cytoplasm was evaluated separately. An immunoreactive score (IRS) based on the percentage of positive cells and staining intensity was applied. The percentage of positive cell scores were assigned according to the following scale: 0: 0%; 1: 1-20%; 2: 21-50%; 3: 51-80%; 4: >80%. Staining intensity was scored semi-quantitatively as follows: 0: none; 1: mild; 2: moderate; and 3: intense. A total score for each membrane and cytoplasmic staining was then obtained (ranging from 0 to 12). Results were summed up and divided by the number of evaluation procedures to receive an average.

### Statistical analysis

Data analyses were performed using SPSS (SPSS Inc, Chicago, IL, USA) and GraphPad Prism Software (version 5.0). Fisher’s exact or chi-square tests were used to evaluate differences in clinico-pathological characteristics between AA and CAU women and correlations between expression of ALCAM (low or high) and clinic-pathological characteristics. The association of ALCAM expression with ethnicity, age, histological grade, tumor size, lymph node status, ER/PR status and HER2-neu status was determined by logistic regression with multivariate analysis. Odds ratio (OR), and 95% confidence interval (CI) were also calculated. Two-sided p values were calculated. Differences and correlations were considered significant if p value was < 0.05 (*), <0.01 (**) and <0.001 (***).

## Results

### Clinico-pathological characteristics of AA and CAU breast cancer

Clinico-pathological characteristics of breast cancer patients are summarized in Table 1. Of 173 breast cancer patients, there were 95 CAU and 78 AA women. In both ethnic groups, there was relatively equal distribution of age at diagnosis [diagnosed with breast cancer at age greater than 50 years (CAU 69.5% vs. AA 70.5%; p = 0.99)]. The predominant histological type was ductal adenocarcinoma (CAU 77.8% vs. AA 97.4%; p = 0.003), and there was no statistical difference in histological grade between the two ethnic groups (p = 0.069). AA women presented with a significantly lower proportion of smaller size tumors (T1) compared to CAU women (AA 43.6% vs. CAU 72.6%; p = 0.026). However, no statistical difference was found in clinical staging or nodal status at presentation between the two ethnic groups (Table 1). Breast cancers in AA women were ER Negative in larger proportions (38.5%) compared to the CAU patients (15.8%, p = 0.001), whereas there was no difference in PR expression (p = 0.081). More than three fourths of all patients were HER-2 negative, and there was no significant difference between the AA and CAU patients (p = 0.13). One third of all AA patients showed negative markers for ER, PR and HER-2 neu (triple negative), compared to only 10.5% of CAU patients (p = 0.0008).

**Table 1:** **Clinical and pathological characteristics of AA and CAU breast cancer in Atlanta**

Characteristics	CAU (n = 95)		AA (n = 78)		***p***value
	n	%	n	%	
Age at diagnosis					0.99
≤ 50 years	29	30.5	23	29.5	
> 50 years	66	69.5	55	70.5	
Histological type					0.003*
Ductal	74	77.8	76	97.4	
Lobular	7	7.4	2	2.6	
Ductal/Lobular	7	7.4	0		
Missing	7	7.4			
Histological grade					0.069
G1	29	30.5	16	20.5	
G2	44	46.3	29	37.2	
G3	17	17.9	24	30.8	
Missing	5	5.3	9	11.5	
Tumor size					0.026*
T1	69	72.6	34	43.6	
T2	15	15.8	20	25.6	
T3-T4	6	6.3	7	9.0	
Missing	5	5.3	17	21.8	
Lymph node status					0.68
Negative	48	50.5	38	48.7	
Positive	25	26.3	23	29.5	
Missing	22	23.2	17	21.8	
AJCC stage					0.65
I	40	42.1	28	35.9	
II	22	23.2	18	23.1	
III-IV	11	11.5	12	15.4	
Missing	22	23.2	20	25.6	
ER status					0.001*
Negative	15	15.8	30	38.5	
Positive	70	73.7	45	57.7	
Missing	10	10.5	3	3.8	
PR status					0.081
Negative	28	29.5	34	43.6	
Positive	57	60	41	52.6	
Missing	10	10.5	3	3.8	
HER2-neu status					0.13
Negative	67	70.5	65	83.4	
Positive	19	20	9	11.5	
Missing	9	9.5	4	5.1	
Triple negative					0.0008*
Yes	10	10.5	25	32.0	
No	74	77.9	51	65.4	
Missing	11	11.6	2	2.6	

AJCC: American Joint Committee on Cancer; CAU: Caucasian; AA: African-American; ER/PR: estrogen/progesterone receptor; HER2-NEU: human epidermal growth receptor 2. *Age-adjusted.

### Overall ALCAM expression in AA and CAU breast cancer tumors

**Figure 1:**
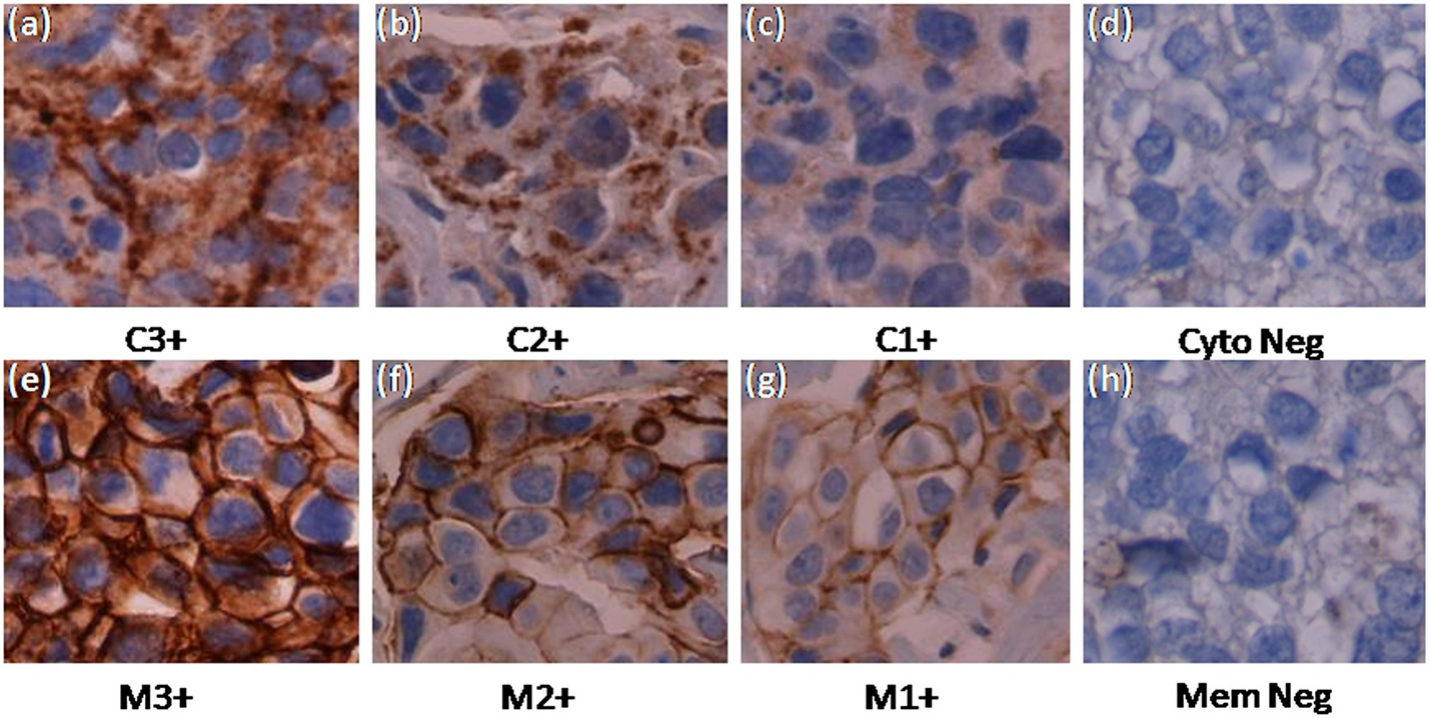
**The intensity of immunohistochemistry staining with ALCAM in breast cancer tissue. (a)** C3+ positive: intensity is very strong in cytoplasmic staining. **(b)** C2+ positive: clear staining but the intensity is not as strong as c3+. **(c)** C1+ positive: some light cytoplasmic staining. **(d)** Negative: no staining. **(e)** M3+ positive: intensity is very strong in membranous staining. **(f)** M2+ positive: clear staining but the intensity is not as strong as M3+. **(g)** M1+ positive: some light membranous staining. **(h)** Negative: no staining. Original magnification, x400.

**Figure 2:**
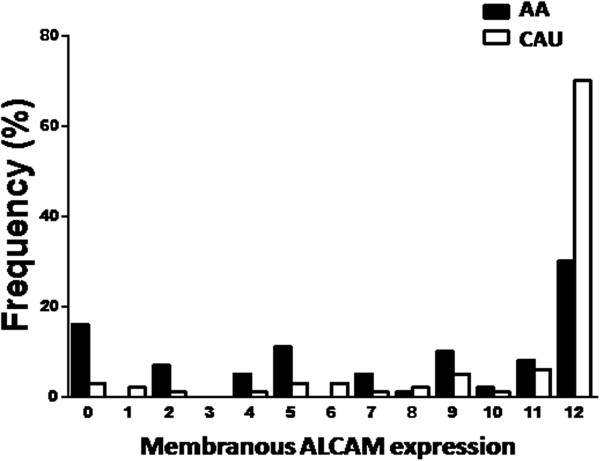
**Distribution of membranous ALCAM expression in AA and CAU breast cancer patients.** ALCAM levels were determined by immunohistochemistry (IHC) according to the immunoreactive Score (IRS) by combining both intensity of IHC and percentage of tumor cells stained as stated in materials and methods. The x-axis indicates the Membranous ALCAM Immunoreactive score (IRS). The range of IRS is from 0 (negative staining) to 12. The y-axis indicates the frequency of cases.

Immunohistochemical analysis of ALCAM was performed to determine its subcellular localization and level of expresssion in primary breast tumors of AA and CAU women. ALCAM was locliazed to intercellualr junctions/membrane and the cytoplasm. The stain intensity ranged from light to dark brown and was scored as 3+ positive (very strong), 2+ positive (clear staining but not as strong as 3+), 1+ positive (some lighter staining), and negative (no staining) (Figure 1). We developed a Immunoreactive Score (IRS) by combining both intensity of IHC and percentage of ALCAM-positive tumor cells. ALCAM expression in CAU and AA breast cancer tissue is listed in Table 2. There were significant differences in membrane ALCAM expression between the two ethnic groups, with a higher proportion of higher scores (IRS 8–12) in CAU as compared to AA (CAU 85.2% vs. AA 53.8%; p < 0.0001) (Figure 2). Notably, there was a higher proportion of negative membranous staining in AA patients as compared to CAU patients (AA 16.7% vs. CAU 3.2%). However, there was no difference in the degree of cytoplasmic ALCAM staining between the two groups (p = 0.06).

**Table 2:** **ALCAM expression in AA and CAU breast cancer**

Ethnicity	Membranous staining n (%)			***p***value	Cytoplasmic staining n (%)		***p***value
	Negative staining	IRS 1-7	IRS 8-12		IRS 1-7	IRS 8-12	
CAU (n = 95)	3(3.2)	11(11.6)	81(85.2)	<0.0001*	29(30.5)	66(69.5)	0.06
AA (n = 78)	13(16.7)	23(29.5)	42(53.8)		35(44.9)	43(55.1)	

CAU: Caucasian, AA: African-American, IRS: immunoreactive score, *Age-adjusted.

### ALCAM and clinical characteristics of AA and CAU breast cancer

We examined membranous ALCAM in the context of patient characteristics and tumor pathology. Poorly differentiated tumors showed lower intensity membranous staining (IRS 0–7) (AA: p = 0.04; CAU: p = 0.02) (Table 3). An increase in membranous ALCAM expression associated with positive ER status (AA: p = 0.0004; CAU: p = 0.0015) and PR status (AA: p = 0.002; CAU: p = 0.034) in both ethnic groups. Most notably triple negative tumors (TN: ER, PR, and HER-2 neu negative) showed significantly lower intensity membranous staining (IRS 0–7) as compared to tumors that were not triple negative in both ethnic groups (AA; p = 0.0002; CAU; p = 0.0006). There was no significant correlation with the membranous pattern of staining and age of the patient, histological tumor type, tumor size, lymph node status or HER2-neu status in either ethnic group.

**Table 3:** **Membranous ALCAM expression and clinical and histological tumor characteristics of AA and CAU breast cancer**

	Membranous ALCAM (n,%) in Caucasian			Membranous ALCAM (n,%) in African American		
	IRS 0-7	IRS 8-12	***p***value	IRS 0-7	IRS 8-12	***p***value
Age in years			0.41			0.65
≤50	3(3.1)	26(27.4)		10(12.8)	13(16.7)	
>50	11(11.6)	55(57.9)		27(34.6)	28(35.9)	
Histological Type			0.38			0.93
IDC	10(11.4)	64(72.7)		36(46.2)	40(51.3)	
ILC	0(0)	7(8)		1(1.3)	1(1.3)	
IDC&ILC	1(1.1)	6(6.8)		0(0)	0(0)	
Histological Grade			0.02*			0.04*
G1	3(3.3)	26(28.9)		5(7.2)	11(15.9)	
G2	3(3.3)	41(45.6)		11(15.9)	18(26.1)	
G3	6(6.7)	11(12.2)		16(23.2)	8(11.6)	
Tumor size			0.71			0.82
T1	9(10)	60(66.7)		15(24.6)	19(31.1)	
T2	3(3.3)	12(13.3)		9(14.8)	11(18.0)	
T3-T4	1(1.1)	5(5.5)		4(6.6)	3(4.9)	
Lymph node status			0.95			0.44
Negative	6(8.2)	42(57.5)		16(26.2)	22(36.1)	
Positive	3(4.1)	22(30.1)		12(19.7)	11(18)	
ER status			0.0015*			0.0004*
Negative	7(8.2)	8(9.4)		21(28)	9(12)	
Positive	6(7.1)	64(75.3)		13(17.3)	32(42.7)	
PR status			0.034*			0.002*
Negative	8(8.9)	20(23.5)		22(29.3)	12(16)	
Positive	5(5.9)	52(61.2)		12(16)	29(38.7)	
HER2-neu status			0.14			0.096
Negative	12(14)	55(63.9)		31(41.9)	34(45.9)	
Positive	1(1.2)	18(20.9)		2(2.7)	7(9.5)	
Triple Negative			0.0006*			0.0002*
Yes	6(7.1)	4(4.8)		19(25)	6(7.9)	
No	7(8.3)	67(79.8)		16(21.1)	35(46.1)	

*Age-adjusted.

### Multivariable analysis of ALCAM expression with ethnicity

Since loss of ALCAM function is a bad prognostic marker in breast cancer, we examined which factors would contribute most significantly to its expression. We built a multivariable logistic regression model to include basal-like subtype, ethnicity and four additional covariates (age, histological grade, tumor size, lymph node status). Table 4 demonstrates that ethnicity contributed significantly to ALCAM expression after adjustment for the other covariates. Compared to CAU, the AA tumors were about 4-times more likely to have low ALCAM expression (p = 0.003), after accounting for basal-like status and the other four variables. Basal-like status was associated with ALCAM expression (p = 0.01) however in this model, age (under or above 50 years), histological grade (poorly and well-moderately), tumor size [large (>2 cm) and small (≤2 cm)], lymph node status, did not contribute to ALCAM expression.

**Table 4:** **Multivariable logistic regression analysis of the association between ethnicity and ALCAM expression in breast cancer**

	Membranous ALCAM level n (%)			
	IRS 0-7	IRS 8-12	Odds ratio (95% CI)	***P***value
Ethnicity				
AA	37 (72.55)	41 (33.61)	4.28(1.64- 11.15)	0.003
CAU	14 (27.45)	81 (66.39)	Reference	
Age in years				
≤50	13 (25.49)	39 (31.97)	0.69 (0.24- 1.99)	0.49
>50	38 (74.51)	83 (68.03)	Reference	
Histological grade				
Poor	22 (50.00)	19 (16.52)	2.10(0.60- 7.34)	0.25
Well-moderately	22 (50.00)	96 (83.48)	Reference	
Tumor size				
Large (>2 cm)	17 (41.46)	31 (28.18)	0.62( 0.20-1.88)	0.39
Small (≤2 cm)	24 (58.54)	79 (71.82)	Reference	
Lymph node				
Positive	15 (40.54)	33 (34.02)	1.62( 0.58- 4.54)	0.36
Negative	22 (59.46)	64 (65.98)	Reference	
Basal-like (TN)				
Yes	25 (51.02)	10 (8.85)	5.59(1.46- 21.50)	0.01
No	24 (48.98)	103 (91.15)	Reference	

## Discussion

ALCAM is emerging as an important molecule in cancer due to its consistent differentiation of aggressive phenotypes, prognosis and response to therapy [19, 23, 26]. The goal of this study was to define for the first time the role of ALCAM in the ethnic disparity of breast cancer in the US. The major findings are that ALCAM expression at the critically important intercellular junctions of primary breast cancer tumors is markedly lower in AA women compared to CAU women, regardless of age, histological grade, tumor size and lymph node involvement, ER/PR and HER2-neu status. These findings suggest that ALCAM may dominantly contribute to the aggressive behavior of breast cancer among AA women.

Breast cancer in AA women is characterized by higher grade, later stage at diagnosis and worse survival rate [2, 27]. Variations in tumor biology at several stages of the disease process likely contribute to this disparity. Molecular and genetic profiling has revealed inter- and intra-ethnic heterogeneity of breast cancer with varied prognoses and responses to therapy [28–30]. Luminal tumors have the most favorable outcome while Her2-overexpressing and basal-like (triple-negative, TN) tumors have the worst prognoses [31–34]. Triple-negative tumors are aggressive with a peak risk of recurrence within three years of diagnosis [35, 36]. This aggressive subtype is more common in AA women [31, 37, 38], and contributes to the poor prognosis in young AA women [31]. We confirmed the major ethnic-related histological and molecular phenotypes of breast cancer in our two populations, which provided an important validation of the ethnic categorization of patients, clinicopathological evaluations and the data analyses we used in our study (Table 1). Even after accounting for this major confounder (basal-like subtype), ALCAM expression at intercellular tumor junctions was significantly lower among the AA women with breast cancer (Table 4). These findings reaffirm the prognostic status of ALCAM in breast cancer.

Ethnic differences in ALCAM expression were found when tumors were characterized by histological grade, size and lymph node involvement. ALCAM is thought to contribute to events that influence the transition of homotypic behavior in tumor masses to heterotypic interactions with surrounding cell types. This process is driven in part by proteolytic cleavage of membrane bound ALCAM by ADAM/TACE and its subsequent loss from intercellular tumor junctions [39]. Cytoplasmic overexpression of ALCAM is prognostically relevant in breast cancer [22]. The mechanism responsible for this association of higher cytoplasmic ALCAM levels with a more aggressive course of the disease is not fully addressed. In the current study, we did not find a significant association of cytoplasmic ALCAM expression with ethnicity. We reported recently that ALCAM dominantly influences the adhesive phenotypes of breast cancer cells in the pulmonary vasculature, a process that influences metastasis to the lung. MDA-MB-231 cells, which cannot metastasize to distant sites when injected into the mammary fat pad of athymic nude mice [40, 41] formed large ALCAM-mediated homotypic intravascular cell clusters in an isolated perfuse rat lung model. Conversely, ALCAM-negative MDA-MB-435 cells that spontaneously form distant metastasis [40–43] formed similar clusters only after ectopic ALCAM transfer [44, 45]. In this study, we found that most AA tumors had low or complete loss of ALCAM expression at intercellular junctions regardless of the basal-like status, level of differentiation, tumor size, lymph node involvement and age (Table 4). Compared to CAU tumors, The AA are about 4 times more likely to have low ALCAM expression (p = 0.003). ALCAM expression was high in virtually all well-differentiated tumors in CAU women while nearly one-third of tumors of the same level of differentiation in AA women had low ALCAM. This discordance revealed that well-differentiated tumors in AA women are equivalent in number to poorly differentiated tumors in CAU women with respect to ALCAM expression.

It is thought that the level of differentiation of breast cancer tumors influences their overall aggressive phenotype. A lower proportion of breast cancer among the AA women in our study was well differentiated as expected (CAU 76.8% vs AA 57.7%). In addition, we uncovered a new source of biological variation (i.e. reducing ALCAM expression), which suggests that well differentiated tumors may adopt a more aggressive phenotype uniquely among AA women. This assertion is based on results showing low ALCAM expression in significantly higher number of well differentiated tumors in this patient population. Overall, our data suggests that compared to CAU women, breast cancer tumors of equivalent size and histological grade in AA women are more likely to lose homotypic adhesions within the tumor mass, which would promote their metastatic potential.

## Conclusions

ALCAM expression at intercellular tumor junctions correlates with tumor grade, ER status, PR status and triple-negative tumor status in breast cancer patients. Down-regulation of ALCAM is more severe in AA women than in CAU women even when the tumors have identical characteristics, such as histological grade, tumor size and lymph involvement. Lower ALCAM expression may contribute to the aggressive phenotype of breast cancer among AA women.

## Abbreviations

ALCAM: Activated leukocyte cell adhesion molecule
AA: African-American
CAU: Caucasian
FFPE: Formalin-fixed paraffin-embedded
IRS: Immunoreactive score
ER: Estrogen receptor
PR: Progesterone receptor
HER2: Human epidermal growth receptor 2
TN: Triple negative.
